# Effectiveness of a socioecological model-guided, smart device-based, self-management-oriented lifestyle intervention in community residents: protocol for a cluster-randomized controlled trial

**DOI:** 10.1186/s12889-023-17073-w

**Published:** 2024-01-02

**Authors:** Shujuan Yang, Bin Yu, Kai Liao, Xu Qiao, Yunzhe Fan, Ming Li, Yuekong Hu, Jiayan Chen, Tingting Ye, Changwei Cai, Chunlan Ma, Tong Pang, Zixing Huang, Peng Jia, Jan D. Reinhardt, Qingyu Dou

**Affiliations:** 1 West China School of Public Health and West China Fourth Hospital, Sichuan University, Chengdu, 610041 China; 2 Department of Health Management Center, Clinical Medical College & Affiliated Hospital, Chengdu University, Chengdu, 610106 China; 3 Respiratory Department, Chengdu Seventh People’s Hospital, Chengdu, 610021 China; 4 International Institute of Spatial Lifecourse Epidemiology (ISLE), Wuhan University, Wuhan, China; 5 Institute for Disaster Management and Reconstruction, Sichuan University, Chengdu, 610207 China; 6 Department of Radiology, West China Hospital, Sichuan University, Chengdu, 610041 China; 7 West China Tianfu Hospital, Sichuan University, Chengdu, 610200 China; 8 School of Public Health & Jiangxi Provincial Key Laboratory of Preventive Medicine, Nanchang University, Nanchang, 330006 China; 9 School of Resource and Environmental Sciences, Wuhan University, Wuhan, 430072 China; 10 Department of Rehabilitation Medicine, Jiangsu Province Hospital/Nanjing Medical University First Affiliated Hospital, Nanjing, 210009 China; 11 Swiss Paraplegic Research, 6207 Nottwil, Switzerland; 12 Department of Health Sciences and Medicine, University of Lucerne, 6002 Lucerne, Switzerland; 13 National Clinical Research Center of Geriatrics, Geriatric Medicine Center, West China Hospital, Sichuan University, Chengdu, 610041 China

**Keywords:** Healthy lifestyles, mHealth, Socioecological model, Smart device, Self-management

## Abstract

**Background:**

Healthy lifestyles are crucial for preventing chronic diseases. Nonetheless, approximately 90% of Chinese community residents regularly engage in at least one unhealthy lifestyle. Mobile smart devices-based health interventions (mHealth) that incorporate theoretical frameworks regarding behavioral change in interaction with the environment may provide an appealing and cost-effective approach for promoting sustainable adaptations of healthier lifestyles. We designed a randomized controlled trial (RCT) to evaluate the effectiveness of a socioecological model-guided, smart device-based, and self-management-oriented lifestyles (3SLIFE) intervention, to promote healthy lifestyles among Chinese community residents.

**Methods:**

This two-arm, parallel, cluster-RCT with a 6-month intervention and 6-month follow-up period foresees to randomize a total of 20 communities/villages from 4 townships in a 1:1 ratio to either intervention or control. Within these communities, a total of at least 256 community residents will be enrolled. The experimental group will receive a multi-level intervention based on the socioecological model supplemented with a multi-dimensional empowerment approach. The control group will receive information only. The primary outcome is the reduction of modifiable unhealthy lifestyles at six months, including smoking, excess alcohol consumption, physical inactivity, unbalanced diet, and overweight/obesity. A reduction by one unhealthy behavior measured with the Healthy Lifestyle Index Score (HLIS) will be considered favorable. Secondary outcomes include reduction of specific unhealthy lifestyles at 3 months, 9 months, and 12 months, and mental health outcomes such as depression measured with PHQ-9, social outcomes such as social support measured with the modified Multidimensional Scale of Perceived Social Support, clinical outcomes such as obesity, and biomedical outcomes such as the development of gut microbiota. Data will be analyzed with mixed effects generalized linear models with family and link function determined by outcome distribution and accounting for clustering of participants in communities.

**Discussion:**

This study will provide evidence concerning the effect of a mHealth intervention that incorporates a behavioral change theoretical framework on cultivating and maintaining healthy lifestyles in community residents. The study will provide insights into research on and application of similar mHealth intervention strategies to promote healthy lifestyles in community populations and settings.

**Trial registration number:**

ChiCTR2300070575. Date of registration: April 17, 2023. https://www.chictr.org.cn/index.aspx.

## Introduction

Healthy Lifestyles, including regular physical activity (PA), balanced diet, smoking cessation, limited alcohol consumption, and healthy body weight, are important contributors to preventing and controlling chronic diseases [1]. In contrast, unhealthy lifestyles are associated with increased risks of cardiovascular and metabolic diseases, as well as mortality [2, 3]. Unfortunately, about 89.6% of Chinese community-resident adults engage in at least one unhealthy lifestyle [2]. Accordingly, there is a need for effective intervention strategies targeting modifiable unhealthy lifestyles.

Traditional community-based intervention studies have typically been implemented at specific geophysical sites [4, 5]. While evidence suggests that such interventions can be effective in promoting and maintaining favorable lifestyles, they can be resource-intensive and costly [6]. Moreover, participants often discontinue participation if they find the interventions unappealing or time-consuming [7, 8]. In comparison, facilitated by the widespread adaptation of smartphones, mobile smart device-based health interventions (mHealth) appear easier to implement on a larger scale and more cost-effective [6, 9]. mHealth interventions can incorporate multidimensional lifestyles management strategies [10], utilizing a range of tools, including text messages, remote health monitoring [10, 11], and web-based programs with digital resources (e.g., videos) [12]. Through timely feedback from participants the strategy itself can furthermore be continuously adapted. Previous randomized controlled trials (RCTs) evaluating mHealth interventions in community dwelling adults have mainly focused on one or two specific lifestyle behaviors (e.g., PA and diet). Behavioral modifications achieved were negligible [13–15], or not sustained [16], in about half of the studies, with the other half demonstrating mostly short term effects [17–19]. One possible reason may be that mHealth interventions for healthy lifestyles are influenced by multi-level environmental factors, including individual, community and policy-level determinants [20–23]. Currently, only a few of community-based RCTs implemented mHealth intervention at multi-level [24, 25]. For example, Tamura and colleges developed a multilevel (individual, work, home, church levels) mHealth PA intervention in resource-limited communities [24]; Hosteng et al., evaluated a mHealth PA intervention that considered individual and social levels [25]. These studies indicated the effectiveness of multilevel mHealth intervention while fail to consider participants’ self-management.

The socioecological model provides a useful theoretical framework that can help to understand how society, community, and individual level factors affect lifestyles and health outcomes of community residents [26, 27]. However, the social-ecological model has also been criticized for lacking an agency dimension that considers people’s decision making and self-management strategies [27, 28]. Empowerment models, in turn, focus on the agency dimension and people’s self-management strategies, emphasizing opportunities to develop self-management skills, social engagement and personalization of goals [29], but neglect the multilayered nature of environmental influences [11]. An integration of empowerment and socio-ecological models may thus be a promising framework to guide smart device-based, and self-management-oriented lifestyle interventions for community residents [30].

The development of mHealth interventions that are guided by the socioecological model and supported by multi-dimension empowerment theory is likely to better promote the adaptation and maintenance of an array of healthy lifestyles, leading to systematic and lifelong health benefits. Therefore, we designed a randomized-controlled community-based trial, named 3SLIFE (i.e., socioecological model-guided, smart device-based, and self-management-oriented lifestyles). The 3SLIFE trial aims to evaluate a mHealth-based intervention that is theoretically supported by empowerment theory and socio-ecological model to promote healthy lifestyles among community residents in China.

## Methods

The protocol has been developed according to the Standard Protocol Items: Recommendations for Interventional Trials (SPIRIT) statement [31] and interventions are described according to the Template for the Intervention Description and Replication (TIDieR) framework [32].

### Study design overview

**Fig. 1:**
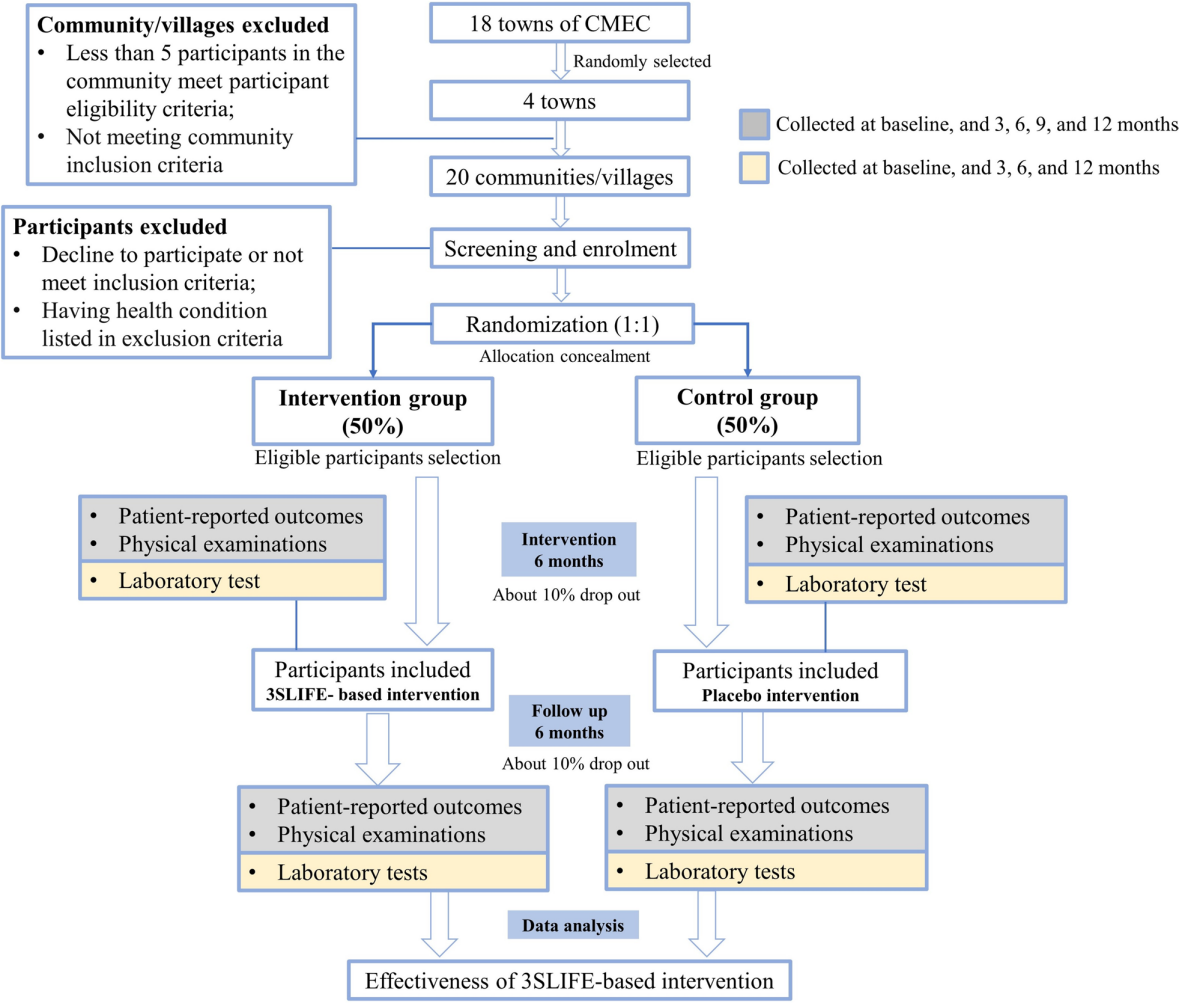
Study design and anticipated participant flow

This is a two-arm, parallel, cluster-RCT targeting the promotion of healthy lifestyle among community residents in China. The study design and intended participant flow through the study is shown in Fig. 1. The study includes a 6-month intervention and a 6-month follow-up phase. We hypothesize that participants in the intervention group will more frequently change unhealthy behaviors towards healthy lifestyle patterns than those in the control group.

### Settings and participants

The study will be embedded in the China Multi-Ethnic Cohort (CMEC) [33], which uses a multistage random sampling strategy to select participants. Four towns (administrative areas in China with about 300,000 inhabitants) will be randomly selected from 18 towns that have undergone two survey waves within the CMEC, and used as the study sites. A total of 20 eligible communities/villages will further be randomly selected within the four towns. These will then be randomized to control or intervention. Eventually, community-dwelling adults from these communities/villages will be screened for eligibility and those who meet the eligible criteria will be invited to participate.

### Eligibility criteria of study sites (community/village) and participants

#### Study sites

##### Inclusion criteria

- Study sites have undergone two survey waves of the CMEC.
- The government of the community/village committee conforms to and agrees with this study protocol;
- No other intervention programs are being implemented in this community/village;
- Distance of at least 2 km to other included villages to minimize contamination risk.

##### Exclusion criteria

- Less than 5 residents of the community in question meet the participant eligibility criteria.

##### Recruitment method

- The community/village is confirmed by the governmental committee of the town (after having been randomly selected), then preliminary screening of candidate participants will be conducted.

#### Participants

##### Inclusion criteria

- Being 18 years old or above;
- Having participated in two survey waves of the CMEC;
- Having access to the internet via a smartphone.

##### Exclusion criteria

- Having a history of major psychiatric illnesses (e.g., schizophrenia and bipolar disorder), Parkinson’s disease, severe cognitive or hearing impairment according to medical records obtained from Sichuan Medical Insurance Bureau.
- Having had major surgery in the past year;
- Receiving cancer treatment;
- Pregnant.

##### Termination criteria

- Withdrawing informed consent and/or withdrawing from the trial;
- Development of new serious diseases that makes it inappropriate to continue the interventions as specified in the trial protocol;
- Experiencing serious adverse events during the intervention phase.

### Randomization and allocation concealment

In this cluster-RCT, recruitment and baseline data collection will be conducted prior to randomization. Cluster randomization, which is necessary for implementation of community-level interventions, mitigates concerns of within-community/village contamination, and improves the feasibility of on-site intervention, will be performed at the community/village level. A total of 20 eligible communities/villages will be randomized using a computer-based random number generator (www.random.org) and assigned to either the intervention group or the control group, with 10 communities/villages in each group. A covariate adaptive procedure will be used to minimize the *P* value from a joint *F* test (1000 iterations) to facilitate covariate balance [34, 35]. Several location level covariates will be considered, including number of village residents, and mean age and sex ratio of residents.

Allocation concealment will be ensured through central randomization [36]. Specifically, an independent statistician will generate a block randomization sequence with randomly permutated block size (4–8) and 1:1 allocation ratio. A sequential list with resulting random numbers will be provided to the study center that recruits villages. Only after participation of a village is confirmed, will the study center reveal labels of the random sequence to the trial team delivering interventions.

### Blinding

Those delivering interventions cannot be blinded to group allocation. Participants, assessors, and statisticians will be blinded throughout the entire trial period. Assessors are independent research assistants who are not involved in intervention delivery. Intervention providers are asked to not disclose group assignment to participants and assessors such as not talking about the mHealth intervention. Statisticians will receive data with unlabeled group coding and labels will only be revealed after completion of statistical analyses and start of interpretation of results.

### Sample size

The primary outcome is the improvement of modifiable unhealthy lifestyles, with a reduction by at least one unhealthy lifestyle at 6 months considered a favorable outcome (coded 1) and any other outcome unfavorable (coded 0). According to a previous study, the prevalence of at least one unhealthy lifestyle in Chinese community residents is 89.6% [2, 37]. According to an analysis of the previous two survey waves of the CMEC, it is expected that 2 percent of the control group participants will achieve a favorable outcome. We assume that a 10% percent increase of participants with favorable outcome in the intervention group (to 12% in total) constitutes a minimally important difference.

To achieve a power *of* 0.80 to obtain a statistically significant signal at α <  = 0.05 for p_1_ = 0.02 vs. p_2_ = 0.12 with a chi-squared test of independence, the sample size is estimated as 204 (102 per group). To account for participant attrition of estimated 20%, recruitment target is 128 participants per group, with a total sample of 256 participants for assessment and evaluation.

### Intervention

#### Intervention model and theory

**Fig. 2:**
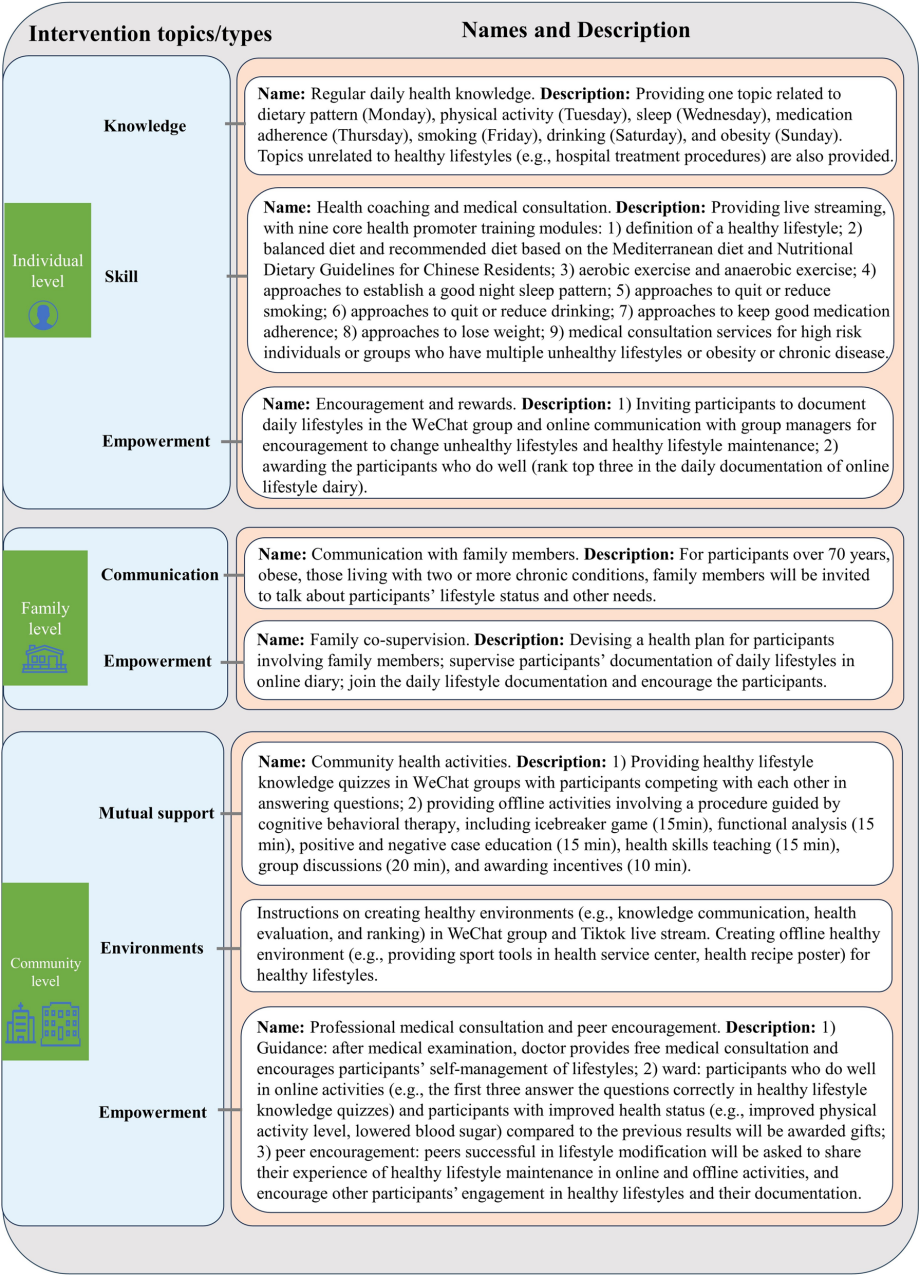
3SLIFE framework outlining the intervention details

The 3SLIFE intervention is primarily based on the framework of the socioecological model and comprises interventions at the individual, family and community levels [38]. Smart device-based and offline interventions will be implemented at each level. Drawing on multi-dimensional empowerment theory, we aim to promote self-management ability among participants at the individual, family and community levels by strengthening self-management skills, fostering social engagement, and personalization of goals [29], rather than relying exclusively on external instruction [39]. Details of the framework are provided in Fig. 2.

#### Intervention design and strategy

The intervention group will receive intervention for the first 6 months after recruitment into the study. In the second 6 months, instructed intervention will be terminated, but self-management of lifestyles can continue based upon participants’ decision and quarterly assessment will continue to observe participants’ maintenance of healthy lifestyles.

According to the 3SLIFE, interventions will take place at three levels: (1) individual level (microsystem): providing medical consultation services and health knowledge, one-to-one health coaching, and requiring participants’ voluntarily documentation of their daily lifestyles; (2) family level (mesosystem): communicating with family members about participants’ lifestyle status, making healthy lifestyle improvement plans with family members, and improving participants’ self-management ability through family supervision; (3) community level (macrosystem): organizing online and offline community activities, creating healthy environments, and improving self-management ability of healthy lifestyles through online healthy lifestyle documentation and offline community activities.

The control group will receive placebo interventions unrelated to healthy lifestyles and chronic disease risk factors (e.g., information about the hospital visit process) for the first 6 months after enrollment. Participants in control group will also complete quarterly assessment. In the second six months, the placebo interventions will be terminated while quarterly assessments continue. To ensure that the control group can benefit from the same healthy lifestyle interventions, all participants in the control group will have access to the same program as the intervention group at the end of the trial followed up with assessments as previously given provision of consent.

More details of intervention strategies are listed [32], in Table 1 and Fig. 2 according to the TIDieR framework [32].

**Table 1:** Intervention strategies for control and intervention groups

	Control group	Intervention group
**Individual**		
Intervention	**·**Content: Providing medical consultation services and knowledge unrelated to healthy lifestyles (e.g., hospital treatment procedures) **·**Material: Homemade videos or weblinks to videos, and pictures **·**Provider: Group manager **·**Where: WeChat group **·**Schedule: For 6 months. Providing topics unrelated to healthy lifestyles twice a week	**Knowledge:** **·**Content: Providing medical consultation services and regular daily health knowledge related to healthy lifestyles and other topics (e.g., hospital treatment procedure) **·**Target: Changing misconceptions about healthy lifestyles; increasing knowledge about healthy lifestyles **·**Material: Homemade videos or weblinks to videos, and pictures **·**Provider: Group manager **·**Where: WeChat group **·**Schedule: For 6 months, providing topics related to healthy lifestyle once a day, and providing topics unrelated to healthy lifestyles twice a week **Skill:** **·**Content: One-on-one health coaching **·**Target: Guiding changes in unhealthy lifestyle; guiding engagement in healthy lifestyles; providing skills in maintaining healthy lifestyles **·**Materials: Live streaming platform, online course (e.g., exercise, diet), recipe (guided by the Mediterranean diet), tools (e.g., fitness aids such as elastic bands, medication aids) **·**Provider: Health professor, group manager **·**Where: WeChat and Tiktok live streaming platform, and homemade videos **·**Schedule: Being scheduled for 6 months. Live classes are offered twice a week; daily recipe and daily exercise guidance were provided
Self-management	None	**Empowerment:** **·**Content: Participants’ voluntarily online documentation of their daily lifestyle **·**Target: Self-management of health lifestyles with encouragement, self-supervision, and supervision from others **·**Materials: Gift (household goods) **·**Where: WeChat and offline activities **·**Provider: Group manager **·**Schedule: Inviting all participants document daily lifestyles (scheduled for 12 months) One-to-one online communication once a week to remind unhealthy lifestyle modification or healthy lifestyle maintenance (within 6 months). Being awarded with gifts consisting of daily necessities (e.g., soap and laundry detergent) for participants who document lifestyles well for that month in monthly offline activity (within 6 months)
**Family**		
Intervention	**·**Content: For highly vulnerable participants (e.g., living with two or more chronic diseases), communicating with family members about participants’ health need unrelated to healthy lifestyle (e.g., healthcare reimbursement) **·**Provider: Group manager and local medical workers **·**Where: WeChat, phone **·**Schedule: For 6 months. Online communication with family members if needed	**Communication:** **·**Content: For highly vulnerable participants (e.g., living with two or more chronic diseases) or those requesting this, inviting family members to learn about participants’ lifestyle status and health needs unrelated to healthy lifestyle **·**Target: Learning about participants’ lifestyles **·**Where: WeChat, phone, and face-to-face communication **·**Provider: Group manager and local medical workers **·**Schedule: For 6 months. Inviting family members to attend monthly offline activities and talking about participants’ health status one to one; online communicating with family members once a month about the participant's lifestyles in the previous month
Self-management	None	**Empowerment** **·**Content: For highly vulnerable participants, development and revision of lifestyles modification plan (e.g., exercise three times a week and documentation); co-supervision **·**Target: Promoting family members’ participation in managing participants’ lifestyles; increasing participants' confidence in maintaining healthy lifestyles **·**Where: WeChat, phone, and face-to-face communication **·**Provider: Group manager and family members **·**Schedule: Making healthy lifestyles plan (e.g., exercise three times a week) in the first month post-intervention; online communicating with family members once a month about the participant's lifestyles (within 6 months). Asking family member to join and help with documentation of daily lifestyles (within 6 months)
**Community**		
Intervention	**·**Content: Holding community health activities unrelated to healthy lifestyles (e.g., infectious disease prevention); providing free medical consultations **·**Provider: Group manager and local medical workers **·**Where: Local medical centers **·**Schedule: For 6 months. Providing offline community activities once a month and offline free medical consultations each quarter.	**Mutual support** **·**Content: Holding online activities (e.g., healthy lifestyle knowledge quizzes) and offline activities related to healthy lifestyle and other topics **·**Target: Increasing interpersonal interaction among participants in the same groups; improving participants’ knowledge and skills of healthy lifestyles **·**Where: WeChat group, local medical centers **·**Materials: Health tools (e.g., fitness aids such as elastic bands, medication aids) **·**Provider: Group manager and local medical workers **·**Schedule: Being scheduled for 6 months. Providing online community activities once a week (healthy lifestyle knowledge quizzes), and providing offline community activities once a month **Environments** **·**Content: Creating a healthy environment **·**Target: Creating environment/atmosphere to motivate participants’ healthy lifestyles **·**Where: WeChat group, local medical centers **·**Materials: Health tools (e.g., fitness aids such as elastic bands, medication aids) **·**Provider: Group manager and local medical workers **·**Schedule: Providing tools and announcing the rules for documentation, rules for awarding prizes as soon as the intervention is underway
Self-management	None	**Empowerment** **·**Content: Medical consultation and peers’ encouragement **·**Target: Developing healthy lifestyle with the help of medical staff and peers who perform well in healthy lifestyles **·**Where: WeChat group, local medical centers **·**Provider: Group manager, doctors (from geriatric department, cardiovascular department, nutrition department, rehabilitation department of top three hospitals), local medical workers, and socially active peers **·**Schedule: Holding offline free medical consultations each quarter, giving advice on healthy lifestyles for participants. Sending gifts to participants who do well in online activities (once a month) and who have improved health status (once a quarter). Encouraging peers who perform well in healthy lifestyles to share their experiences of healthy lifestyle maintenance and encouraging other participants in offline activities, once a month

#### Tools for intervention implementation

Intervention and control groups in each community will be assigned one trained group manager and one local medical doctor or nurse with at least 5 years professional experience. The 3SLIFE intervention will be implemented through online and offline activities. Online interventions will be delivered through WeChat and Tiktok Channels, two mainstream live streaming apps in China, to guide diet, exercise, and medication, etc. For online activities, group manager and local physicians will provide healthy lifestyle information and skills (e.g., exercise guidance, recipes) as well as self-management skills with the support of health tools (e.g., fitness aids such as elastic bands) in WeChat groups and one-on-one communication by WeChat or phone. Offline interventions will be guided by cognitive behavior therapy (CBT) [40] at local health centers through group managers, with group managers and physicians designing and recording related healthy lifestyle promotion activities. During the intervention, investigators will record participants' responses and satisfaction with the intervention and obtain their daily health records (e.g., photos of meal preparation following recommended recipes). For individuals with good adherence to the intervention or improved physical examination indicators, value-added gifts (e.g., household goods) will be released as rewards.

### Data collection and outcomes

Questionnaires and scales will be administered in onsite face to face interviews at baseline, 3, 6, 9, and 12 months. Face to face interviews will be conducted by trained research assistants (students). Physical examinations will be conducted by community doctors with at least 5 years of clinical experience. Biological samples such as blood and stool samples will be collected by trained nurses and delivered to specialized laboratories with procedures to keep the cooling chain and avoid contamination.

#### Primary outcomes

The primary outcome is the reduction of modifiable unhealthy lifestyles immediately after intervention (6 months). Unhealthy lifestyles considered comprise smoking, excess alcohol consumption, physical inactivity, unbalanced diet, and overweight/obesity (representing balance between energy intake and energy expenditure). Items as formulated in the Healthy Lifestyle Index Score (HLIS) from the European Prospective Investigation into Cancer and Nutrition cohort study [41], will be used to measure these lifestyles (see Table 2). Assessment of body mass index (BMI) and categorization into overweight/obesity will rely on relevant recommendations according to Chinese guidelines [42], while dietary index is based on the Mediterranean Diet Score [43]. For reasons of simplicity each lifestyle will be coded as a dichotomous variable with 0 indicating absence, 1 indicating presence of a particular unhealthy lifestyle. The decrease of unhealthy lifestyles by at least one at 6 months is considered favorable.

**Table 2:** Components of lifestyle risk score

Components	Score	Measurement	Definitions in the study
Smoking status	0–4	Questionnaire • History of smoking in the past	Never = 4, ex-smokers quit > 10 years = 3, ex-smokers quit ≤ 10 years = 2, current ≤ 15 cigarettes/day = 1, current > 15 cigarettes/day = 0 [41]
Alcohol intake	0–4	Questionnaire • Alcoholic beverage consumption per day	low consumption of alcohol (< 6.0 g/day = 4, 6.0–11.9 g/day = 3, 12.0–23.9 g/day = 2, 24.0–59.9 g/day = 1, 60 + g/day = 0) [41, 44]
Physical activity	0–4	International Physical Activity Questionnaire • Number of hours per week spent in moderate to vigorous activities, vigorous-intensity PA	Metabolic equivalent tasks for four activity domains (occupational PA; transportation PA; PA for housework; sports and leisure-time PA): fifth quintile = 4, fourth quintile = 3, third quintile = 2, second quintile = 1, first quintile = 0 [41]
Dietary factors	0–4	Food Frequency Questionnaire • MDS, a linear score that incorporates 9 nutritional components of the Mediterranean diet [45]	Scoring 4 to 0 points for top to bottom quintile of the MDS: fifth quintile = 4, fourth quintile = 3, third quintile = 2, second quintile = 1, first quintile = 0 [44]
Overweight/obesity	0–4	Medical examination • BMI	< 22 = 4, 22–23.9 = 3, 24–25.9 = 2, 26–27.9 = 1, ≥ 28 = 0 [46]
Total score	0–20	Total of unweighted five components	

*BMI* Body Mass Index, *MDS* Mediterranean Diet Score, *PA* Physical activity

#### Secondary outcomes

Secondary outcomes include modifications of unhealthy lifestyles at 3, 9, and 12 months, and modifications of specific lifestyles (also including smoking, alcohol consumption, physical activity, diet, and BMI), sleep quality, drug intake adherence, smoking/drinking quitting, mental health outcomes, cognitive function, self-efficacy, implicit health attitudes, social support, frailty, and clinical outcomes at baseline, 3, 6, 9 and 12 months, with details as follows:

##### Sleep quality

Sleep quality will be measured with the Pittsburgh Sleep Quality Index (PSQI) [47].

##### Drug intake adherence

Drug intake adherence will be monitored by a self-designed scale to ask about the time of the most recent missed dose and medication-related behaviors, with reference to Medication Adherence Rating Scale (MARS) [48, 49].

##### Smoking/Drinking carving and quitting

Smoking/Drinking craving will be measured with the Visual Analogue Scale (VAS) and the Penn Alcohol Craving Scale (for smoking, with replacement of “alcohol” by “smoking”) [50]. Self-reported smoking or alcohol consumption uses the past seven days as reference period.

##### Mental health outcomes

Anxiety will be evaluated with the 7-item scale for General Anxiety Disorder (GAD-7) [51], and depression will be evaluated with the Patient Health Questionnaire Depression Module (PHQ-9) [52].

##### Cognitive function

Cognitive function will be measured with the Montreal Cognitive Assessment, Basic Version (MoCA-B) [53].

##### Self-efficacy

Self-efficacy will be measured using the General Self-Efficacy Scale (GSES) [54].

##### Implicit health attitudes

Implicit health attitudes will be measured according to the lay theories of healthy behavior intentions scale [55].

##### Social support

The modified Multidimensional Scale of Perceived Social Support (MSPSS) will be used to assess social support [56].

##### Frailty

Frailty will be assessed with the Phenotype of Frailty scale [57].

##### Clinical outcomes

Development of clinical outcomes related to lifestyles including general obesity, central obesity, hypertension, diabetes mellitus, dyslipidemia, sarcopenia, arteriosclerosis, osteoporosis, and fatty liver, will be compared with past medical history supplemented by physical examination and laboratory test indicators. Venous blood samples, collected after overnight fasting (at least 8 h), will be used for clinical laboratory testing, including routine blood tests, fasting blood glucose, lipid levels and hepatic function. If local clinical centers are not equipped with relevant testing devices, uniform devices will be provided and doctors and nurses will be trained to operate these devices, including bone mineral density densitometers (OSTEOKJ3000) among others. All procedures for clinical examinations will be in line with the standard operating procedures (SOPs) of the CMEC as documented elsewhere [33].

##### Metagenomics and metabolome

Gut microbes and their metabolites related to lifestyles will be detected using metagenomics and metabolomics. Respondents will be asked to collect 5-10 g of their own mid-morning stool on days of onsite assessments using a fecal collection cup issued in advance, that is then handed over to the field staff. After handover, it will be immediately checked and tested for quality, labeled, refrigerated in a 5L ice box and transported to a -80 °C refrigerator for temporary storage on the same day. The stool will be delivered to a Third-Party Medical Laboratory for multi-omics analysis. The procedure of sample collection and storage follows the SOPs of the CMEC as documented previously [33].

### Timeline of data collection

**Fig. 3:**
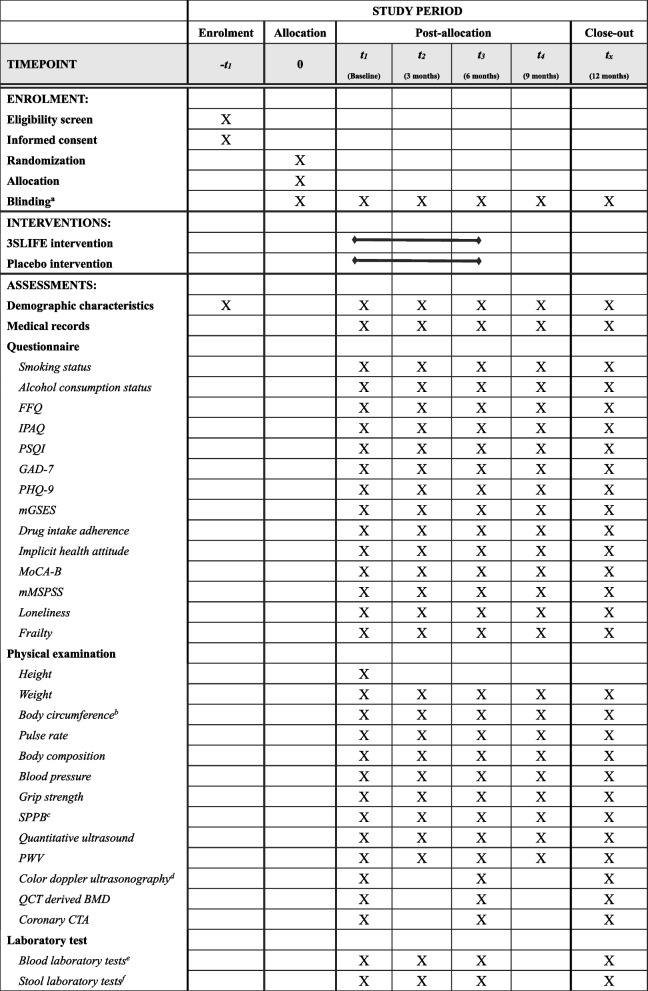
SPIRIT flow diagram for the schedule of enrolment, interventions, and assessments. FFQ, Food Frequency Questionnaire; IPAQ, short version of the International Physical Activity Questionnaire; PSQI, Pittsburgh Sleep Quality Index; GAD-7, 7-item scale for General Anxiety Disorder; PHQ-9, patient health questionnaire depression module; mGSES, modified General Self-Efficacy Scale; MoCA-B, Montreal Cognitive Assessment, Basic Version; mMSPSS, modified Multidimensional Scale of Perceived Social Support; SPPB: Short Physical Performance Battery; PWV, pulse wave velocity; QCT: Quantitative Computed Tomography; BMD: bone mineral density; CTA: computed tomography angiography. ^a^Blinding of participants, assessors, and statisticians. ^b^Body circumference included neck, waist, belly, hip, arm and leg circumference. ^c^SPPB included feet together test, semi-tandem stand test, tandem stand test, 4-m walk test, and five times sit-to-stand test. ^d^Color doppler ultrasonography included abdominal, carotid and cardiac ultrasonography. ^e^Blood tests include total white blood cell count (WBC), red blood cells (RBC), haemoglobin (HGB), platelets (PLT), lymphocytes, monocytes, neutrophils, eosinophils, fasting blood glucose fasting blood glucose (FBG), triglycerides (TG), cholesterol (CHOL), high-density lipoprotein cholesterol (HDL-CH) and low-density lipoprotein cholesterol (LDL-CH), total protein (TP), albumin, globulin, alanine transaminase (ALT), aspartate amino transferase (AST), gamma-glutamyl transpeptidase (GGT), Creatinine (Cr), urea, uric acid (UA) and C-reactive protein (CRP). ^f^Including metagenomics and metabolomics. X The item will be surveyed implemented or on relevant time point.  The item will be implemented in the relevant time period.

Participants who meet the inclusion criteria will undergo baseline data collection given informed consent. Communities will only be randomized after this baseline data collection, with corresponding allocation of participants to experimental or control group. Demographic characteristics and medical records data will respectively be surveyed and extracted at baseline. Primary outcome and secondary outcomes assessed with questionnaires and scales will be collected at five measurement points: baseline, 3 months after baseline, primary end-point. That is end of instructed intervention period (6-month follow-up), 9-month follow-up, and 12-month follow-up. Laboratory data will be collected at four time points: baseline, 3 months, primary end-point (6-month), and 12-month follow-up (Fig. 3).

### Data analysis

Data analyses will be performed using R version 4.2.1 for Windows. All data will be securely stored in an electronic database and accessible only to researchers who meet the criteria as provided in the research ethics approval document.

Descriptive statistics will be provided for the baseline sample. Categorical variables will be summarized in terms of frequencies and percentage, in total and by intervention group. Continues variables will be summarized as mean ± standard or median (interquartile range) in total and by intervention group.

All main analyses will be performed on intention to treat (ITT) basis. The primary outcome will be analyzed with mixed effects log-linear Poisson regression of any reduction of unhealthy lifestyles at six months (favorable outcome vs not) on intervention group as randomized at baseline [58]. In addition, the model will include a random intercept for subject nested in village and a fixed effect for town (as number of clusters not sufficient for variance estimation in the latter case). Treatment effect at six months (immediately after intervention) will be provided as relative risk ratio [59]. As regards secondary outcomes, reduction of unhealthy lifestyles (favorable outcome vs. not) at all timepoints will be simultaneously analyzed with longitudinal mixed effects log-linear Poisson regression on timepoint, and an interaction term of timepoint and intervention group, with random intercepts for subject nested in village and fixed effect for town as specified for the primary outcome. Other binary outcomes including smoking/drinking quitting, drug intake adherence, frailty will be analyzed in the same fashion. For continuous outcomes including sleep quality, smoking/drinking carving, mental health outcomes, cognitive function, self-efficacy, implicit health attitude, social support, longitudinal analysis of covariance (ANCOVA) models with time and time-group interaction will be estimated, adjusted for the respective outcome at baseline and featuring random intercepts for subject nested in village as well as a fixed effect for town [60]. Estimates of differences in changes in lifestyles between groups at 3, 6, 9, and 12 months will be analyzed post hoc using Wald-tests.

#### Missing values

Attrition and dropout rates will be reported in terms of frequencies and percentages and patterns of missing data will be analyzed and respective tables and illustrations provided. Multiple imputation on 100 sets using chained equations with models according to variables’ scale levels will be performed for all main analyses [61].

#### Sensitivity analysis

Results for unimputed data will be reported and results of models adjusted for age and gender. Per-protocol analysis including only those who completed the treatment as planned (at least 70 percent of sessions) will also be performed [62]. Considering the likelihood of missing not at random (MNAR) in mHealth studies, we will also use reference group based imputation for establishing the impact of differential attrition on study results assuming that participants who dropped out during the study behaved like control afterwards [63].

#### Subgroup analysis

We will conduct exploratory subgroup analyses by sex, age, and drug intake adherence. To explore heterogeneity of intervention effects by subgroup, we will include an interaction term between intervention assignment and subgroup in models, and statistical significance will be determined with Wald tests.

### Data monitoring

An independent academic supervisory committee of the CMEC will monitor the proper conduct of the trial and participant safety. During the RCT, participant’s interest will be overseen and safeguarded, the overall conduct of the trial will be monitored, and participants’ safety will be ensured by systematically checking negative events and reacting to any extreme distress. Additional help and counseling will be strongly recommended to participants in the case of any adverse event or emergency.

Periodical meetings will be held after each assessment point to ensure the proper progress of intervention and data collection.

Important protocol modifications will be communicated to relevant parties (i.e., supervisory committee, trial participants, trial registries, ethical committee, and researchers).

### Harms

An adverse event is defined as any unfavorable medical event that occurs after a subject has received an intervention, but is not necessarily related to the intervention. If the current state of the study subject is a pre-existing condition known from medical history, it should not be considered an adverse event unless there is a change in nature, severity, and association. Although the intervention is non-invasive, participants will be asked to inform research assistants as soon as they experience symptoms of discomfort. Any adverse events will be documented as to the nature of the event, time of occurrence, etc., and will be followed up until medically resolved. This study does not involve invasive manipulation or medication, and adverse events expected to occur may be related to the study participant's own illness or the trial. In the course of the trial, in the event of a serious adverse event, the first relevant treatment should be carried out immediately so that the impact of the adverse event on the study subject is minimized; if the interests of the patient conflict with the testing process, the interests of the patient are prioritized, and the trial is terminated if necessary. Study subjects may discontinue or withdraw from the trial at any time or report to the research team. Research assistant will keep a detailed record of all adverse events during the trial, including time, symptoms, and treatment, and report them to the Supervisory Group, the Ethics Committee, and the study sponsor.

### Quality control and quality assurance

All assessors will receive online training (videoconference) as regards the study protocol, including ethical norms and detailed instructions with respect to the evaluation process. Detailed description of assessment procedure will be provided in a standard operation manual. All group managers will receive unified training and learn about experimental and control group interventions before group assignment. Health professionals from local health community hospitals will help scheduling participants to take part in the program and resolve any issues that may arise during the implementation of the intervention.

During the intervention period, a monthly meeting will be held to review the current implementation of the program. Moreover, a series of forms will be designed to document implementation and monitor participants’ safety and compliance, including a form for participant risk grading, and a diary of participants’ adherence to the intervention, with which we will be able to identify individuals at risk and engage in timely activism to improve or enhance interventions.

Quality control (QC) for interviews using scales and questionnaires includes data quality control through inspectors, who will check for outliers and human error on the same day of data collection. QC for medical examination includes checking whether the measurement instruments were in good condition and inspecting whether the measurement was in accordance with standard operating procedures (SOPs). QC for clinical laboratory tests includes sending blind panels of samples to additional Third-Party Medical Laboratories for QC testing. As for QC for data entry, two independent assistants will check the data entered electronically against the trial record sheets. Any data identified as unqualified will be excluded from final analysis. All qualified data is stored on a dedicated data platform before analysis.

### Ethics approval, protocol amendments and consent

This trial will be conducted in compliance with the study protocol, the Declaration of Helsinki, and good clinical practice. Ethical approval for this study has been obtained from the Ethics Committee of the West China School of Public Health and West China Fourth Hospital (Gwll2022096).

The trial has been registered with the Chinese Clinical Trials Registry (ChiCTR2300070575, https://www.chictr.org.cn/index.aspx). The design and implementation of this cluster-RCT is in line with the extension of the SPIRIT 2013 statement [31], and reporting of the trial will be conducted according to the CONSORT statement [64].

Prior to participation, interested participants will be provided with a written informed consent form that includes details of the trial, an explanation of potential risks and benefits, and contact information for study members. Participants may choose not to participate in this study or request to withdraw from the study at any time by notifying the investigator, and any rights of the withdrawing person will not be affected as a result. The investigator may terminate a subject's continued participation in this study if the subject does not comply with the study plan, or if a study-related injury occurs.

### Confidentiality

Questionnaires and sample collection will be undertaken by medical staff, and confidential information will be maintained. The responsible investigator and other investigators will use de-identified names when conducting intervention and analyzing the data and use ID numbers to match results. When the results of this study are published, no individual information will be disclosed.

During the intervention phase, the online intervention will be based on WeChat, the WeChat ID of participants will use ID numbers to replace personal privacy information to ensure information security. All members of the research team need to sign a confidentiality agreement to ensure data security.

## Discussion

Evidence suggests that approximately 90% of Chinese and over 90% of European adults exhibit at least on unhealthy lifestyle behavior including smoking, alcohol consumption, physical inactivity, unbalanced diet and obesity, with higher prevalence found for adults living in less economically developed areas [2, 65]. These unhealthy lifestyles often cluster and have additive or synergistic effects, increasing the risk of chronic disease and multimorbidity [66]. Recent advances in mHealth provide an appealing and cost-effective approach to intervention targeting the reduction of unhealthy lifestyles. To date, only a few mHealth studies using multiple-level lifestyle interventions at multi-levels have been conducted in community-based population, all in the USA [24, 25, 67]. So far respective studies in Chinese community residents have not been reported.

The 3SLIFE trial aims to provide a multi-level theoretical framework for mhealth interventions to develop and maintain multi-dimensional healthy lifestyles in Chinese community residents. Several strength warrant mentioning: First, since lifestyles are often clustered, and in additive or synergistic manner increase the risk of multiple chronic diseases, a multi-dimensional healthy lifestyle intervention, which considers the five main unhealthy lifestyles as suggested by previous large-sample size studies, may be more conducive to overall and sustained health outcomes [37, 68, 69]. Second, the strategies of 3SLIFE will be guided by socioecological model and supported by multi-dimensional empowerment approach, which may overcome the shortcomings of both approaches when taken alone, that is lack of an agency dimension in the socioecological model and lack of a clear multi-level environmental dimension in the empowerment approach. Third, several approaches based on multi-dimensional empowerment will be used to promote self-management-oriented lifestyle changes, such as participants’ voluntarily documentation of their daily lifestyle in WeChat groups, and documentation encouraged by family members or medical staff, which will determine the mHealth adoption and utilization as well as lifestyle changes and sustainability. Fourth, the strategies of 3SLIFE will combine online and offline approaches for lifestyle intervention, which may help participants to become more familiar with smart devices and social media apps and increase trust between participants and medical staff. Fifth, this study will conduct a 6-month follow-up, to evaluate sustainability of intervention effects.

This study has the following limitations we are aware of at this point. First, although smartphones have a high coverage rate in China, some people (such as the elderly) may not be able to use them well, which may hinder the implementation and reduce the effectiveness of mHealth interventions. For example, the present study plans to use the apps WeChat and Tiktok as information resource transmission carrier, which may not be the most attractive tools for older people. However, offline interventions will include instructions on the use of these apps for promoting lifestyle changes. Second, for our primary outcome (lifestyles) and secondary outcomes (such as cognition), we mainly use questionnaire indicators, which may be subject to recall bias and socially desirable answer patterns. However, for some indicators prone to recall bias, we will provide documentation records for participants to help recall their lifestyles. Moreover, all group managers will receive standard investigation training (e.g., using sensitive and appropriate language when speaking about healthy lifestyles and chronic disease) and face to face offline investigation will be conducted in a private room. Third, due to the lack of balanced stratification, comparative analyses between subgroups may not be suitable if the sample size of each group is insufficient across groups (e.g., compare specific lifestyle factors across different types of chronic medical conditions).

Despite these limitations, the 3SLIFE trial presents an initial attempt at systematically promoting evidence-based community health promotion in China and is an important first step towards improved public health research, practice, and policy.

## Data Availability

All investigators and implementation staff will have unrestricted access to the full data set for verification interpretation purposes. Data will be made available to the public after publication of study findings upon request from the corresponding author (Dr. Shujuan Yang, rekiny@126.com; Dr. Qingyu Dou, ddqqking@126.com).
